# Evidence That Transition from Health to Psychotic Disorder Can Be Traced to Semi-Ubiquitous Environmental Effects Operating against Background Genetic Risk

**DOI:** 10.1371/journal.pone.0076690

**Published:** 2013-11-06

**Authors:** Martine van Nierop, Mayke Janssens, Genetic Risk OUtcome of Psychosis (GROUP) Investigators, Richard Bruggeman, Wiepke Cahn, Lieuwe de Haan, René S. Kahn, Carin J. Meijer, Inez Myin-Germeys, Jim van Os, Durk Wiersma

**Affiliations:** 1 Maastricht University Medical Centre, South Limburg Mental Health Research and Teaching Network, EURON, Maastricht, The Netherlands; 2 University Medical Center Groningen, Department of Psychiatry, Groningen, The Netherlands; 3 University Medical Center Utrecht, Department of Psychiatry, Rudolf Magnus Institute of Neuroscience, Utrecht, The Netherlands; 4 Academic Medical Centre, University of Amsterdam, Department of Psychiatry, Amsterdam, The Netherlands; 5 King's College London, King's Health Partners, Department of Psychosis Studies, Institute of Psychiatry, London, United Kingdom; The Nathan Kline Institute, United States of America

## Abstract

**Background:**

In order to assess the importance of environmental and genetic risk on transition from health to psychotic disorder, a prospective study of individuals at average (n = 462) and high genetic risk (n = 810) was conducted.

**Method:**

A three-year cohort study examined the rate of transition to psychotic disorder. Binary measures indexing environmental exposure (combining urban birth, cannabis use, ethnicity and childhood trauma) and proxy genetic risk (high-risk sibling status) were used to model transition.

**Results:**

The majority of high-risk siblings (68%) and healthy comparison subjects (60%) had been exposed to one or more environmental risks. The risk of transition in siblings (n = 9, 1.1%) was higher than the risk in healthy comparison subjects (n = 2, 0.4%; OR_adj_  = 2.2,95%CI:5–10.3). All transitions (100%) were associated with environmental exposure, compared to 65% of non-transitions (p = 0.014), with the greatest effects for childhood trauma (OR_adj_  = 34.4,95%CI:4.4–267.4), cannabis use (OR = 4.1,95%CI:1.1, 15.4), minority ethnic group (OR = 3.8,95%CI:1.2,12.8) and urban birth (OR = 3.7,95%CI:0.9,15.4). The proportion of transitions in the population attributable to environmental and genetic risk ranged from 28% for minority ethnic group, 45% for urban birth, 57% for cannabis use, 86% for childhood trauma, and 50% for high-risk sibling status. Nine out of 11 transitions (82%) were exposed to both genetic and environmental risk, compared to only 43% of non-transitions (p = 0.03).

**Conclusion:**

Environmental risk associated with transition to psychotic disorder is semi-ubiquitous regardless of genetic high risk status. Careful prospective documentation suggests most transitions can be attributed to powerful environmental effects that become detectable when analysed against elevated background genetic risk, indicating gene-environment interaction.

## Introduction

Several environmental factors have been implicated in the aetiology of psychotic illness including urban birth and upbringing [1], minority position [2], childhood trauma [3] and cannabis use [4]. The impact of some environmental risk factors may be greater in those at increased genetic risk suggesting possible interaction between genetic and environmental risks [5].

Associations between environment and mental illness typically are assessed in case-control studies that are easy to conduct but prone to bias and confounding. Cohort studies following individuals from health to illness transition are preferable but expensive and impractical given long incubation periods between exposure and outcome. In addition, given that the prevalence of psychotic disorder in the general population is low [6], cohort studies require large samples that, in order to reduce costs, are subjected to inaccurate non-clinical diagnostic assessments. A high-risk cohort study, following individuals with higher than average genetic risk for psychotic disorder has advantages, given that transition rates will be higher, thus reducing required length of follow-up. In addition, by combining individuals of high average risk in the cohort, a proxy variable indexing genetic risk is created allowing for additional examination of genetic effects, as well as gene-environment interaction [7]. There is also research on transition in individuals described at ‘Ultra-High Risk’, however transition in this population does not refer to transition from health to psychotic disorder, as UHR samples in fact already are help-seeking patients with mental disorder diagnoses [8] who present for treatment at mental health services [9]. In the current article, a detailed and careful follow-up was conducted of healthy siblings (of patients with non-affective psychotic disorder) and healthy comparison subjects sampled in the context of the GROUP study [10], in order to determine true transitions from (non-psychotic) health to psychotic disorder on the basis of clinical interview, and determine the contribution of genetic and environmental factors, taking into account the range of postnatal risk factors for which meta-analytic evidence exists [5]. Given these findings, we expect that urban birth, minority position, childhood trauma and cannabis use all contribute to an increased probability of transition to psychotic disorder, and that this impact may be increased in individuals at higher than average genetic risk.

## Methods

### Subjects

Full details of the GROUP study have been presented elsewhere [10], [11]. In representative geographical areas in the Netherlands and Belgium, patients were identified through clinicians working in regional psychotic disorder services, whose caseload was screened for inclusion criteria. Subsequently, a group of patients presenting at these services either as out-patients or in-patients were recruited for the study. Healthy comparison subjects were selected through random mailings to addresses in the catchment areas of the cases. The GROUP study was not conducted in a geographically well- defined small area, as it in fact included the majority of mental health services in the Netherlands, and a substantial part of mental health services in Dutch-speaking Belgium. Healthy comparison subjects could not be representative in all aspects, as an exclusion criterion was absence of a family history of psychotic disorder. The goal was to collect a control group that (i) was collected from the same geographical area as the case in the relevant mental health service, (ii) was sufficiently large to allow for chance variation and (iii) was frequency-matched in age- and sex distribution to the siblings and (iv) had absence of family history of psychotic disorder. Table 1 shows that healthy comparison subjects and siblings had similar sex distribution and also did not have large differences in age.

**Table 1:** Demographics of participants in the GROUP study.

Variable	Siblings (n = 1057)	Healthy comparison subjects (n = 589)	
	Mean (standard deviation)	Mean (standard deviation)	t value (p)
Age at T0	27.8 (8.3)	30.4 (10.6)	5.53 (<0.001)
Gender, male (%)	45.6	45.7	−0.03 (0.511)
Education, Verhage^a^	5.1 (2.1)	5.4 (1.8)	3.26 (0,001)
WAIS-III Estimated IQ	103.0 (15.3)	109.9 (14.8)	8.73 (<0.001)
Ethnicity, Caucasian (%)	83.2	92.0	4.98 (<0.001)
Urbanicity at birth^b^	2.7 (1.7)	2.6 (1.7)	−0.27 (0.790)

a Education (Verhage): range 0 (no education), 3–5 (school diploma) to 8 (university degree).

b Urbanicity: 1 = <500/km2; 2 = 500–1000/km2; 3 = 1000–1500/km2; 4 = 1500–2500/km2; 5 = 2500+/km2.

The full GROUP sample at baseline consisted of 1119 patients with non-affective psychotic disorder, 1057 siblings of these patients, 919 parents of the patients and 589 unrelated healthy comparison subjects. Inclusion criteria were: (i) age range 16 to 50 years and (ii) good command of Dutch language. For patients, an additional inclusion criterion was the presence of a clinical diagnosis of non-affective psychotic disorder. Healthy comparison subjects status was confirmed by using the Family Interview for Genetic studies [12] with the control as informant, to establish absence of first degree relatives with a psychotic disorder. Diagnosis was based on the Diagnostic and Statistical Manual of Mental Disorder-IV (DSM-IV) criteria [13], assessed with the Comprehensive Assessment of Symptoms and History (CASH) interview [14] or Schedules for Clinical Assessment for Neuropsychiatry (SCAN 2.1) [15]. The majority of patients had a DSM-IV diagnosis of schizophrenia (DSM-IV 295.x; n = 940, 84%). In the sibling and control groups, there were respectively 151 (14%) and 60 participants (10%) with a history of a common mental disorder at baseline, the majority of whom had a mood disorder (DSM-IV 296.x). For the purpose of the current analysis, the siblings and healthy comparison subjects groups were included.

The study was approved by the standing ethics committee (Medisch Ethische Toetsingscommissie, UMC Utrecht), and all the subjects gave written informed consent in accordance with the committee's guidelines. This committee waived the need for additional informed consent of parents or supervisors for underaged participants ages 16 and older, given the non-experimental/medical nature of this study.

### Substance use

Substance use was assessed using the Composite International Diagnostic Interview (CIDI) [16] and through urinalysis. Two different measures of cannabis exposure, assessed both at baseline and follow-up were used to construct incident exposure to cannabis over the follow-up period: (i) CIDI lifetime cannabis use (hereafter: *interview cannabis use*): none (0), versus any use (1) and (ii) current cannabis use assessed by urinalysis (hereafter: *urinalysis cannabis use*): none (0) and present (1). Urinalysis was carried out as a screen for the presence of cannabis at the national Alcohol- and Drug use ‘Jellinek’ Laboratory. The method used was immunoassays with a cut-off of 50 ng/ml. In addition, as an integrity parameter, the creatinine level of every sample was measured. Cannabis urine screening has a detection window up to 30 days, but the detection time has been documented in literature to be even longer (up to three months), depending on level of cannabis use [17]. Given the relatively high cut-off level of 50 ng/ml, a conservative detection window of one month can be inferred. A dichotomous measure was created reflecting first exposure to cannabis over the follow-up period, defined as any instance of positive *interview cannabis use* or positive *urinalysis cannabis use* in those without *interview cannabis use* and without *urinalysis cannabis use* at baseline.

### Childhood trauma

Childhood trauma was assessed with the Dutch version of the Childhood Trauma Questionnaire (CTQ) 25 item Short Form [18], consisting of 25 items rated on a 5-point Likert scale (1 =  never to 5 =  very often). Emotional, physical and general abuse, and emotional and physical neglect were assessed, five items covering each trauma type [18]. Total trauma represents the mean score of all 25 items (range in siblings and healthy comparison subjects: 1–4.3). Conform previous analyses in this sample [19], trauma scores were dichotomized *a priori* into high trauma and low trauma, the cut-off being defined as the 80th percentile of scores for the healthy comparison subjects.

### Urban birth

Subjects were asked where they were born. To describe urbanicity, a historical population density record was generated for each municipality from 1930 onwards using the database of the Central Bureau of Statistics (Netherlands) and the HISSTAT database (University of Gent, Department Modern History, Belgium). When data was not available, linear extrapolations were computed. When historical names of municipalities disappeared from historical records (e.g. due to city mergers) available date from the agglomerate city were used. For each location, population density (by square kilometre, excluding water) at the municipality for that year was computed, on the basis of which the urbanicity code (1 = <500/km2; 2 = 500–1000/km2; 3 = 1000–1500/km2; 4 = 1500–2500/km2; 5 = 2500+/km2) was calculated. In accordance with research using the five-level exposure, a binary urban birth exposure was calculated combining categories 1 to 3 (“0”), reflecting low urbanicity, and 4 and 5 (“1”), reflecting high urbanicity [20], [21].

### Psychosis measures

The Community Assessment of Psychic Experiences (CAPE; www.cape42.homestead.com) was developed in order to rate self-reports of lifetime psychotic experiences. Items are modelled on patient experiences as contained in the PSE-9 [22] and the schedules assessing negative symptoms such as the Scale for the Assessment of Negative Symptoms (SANS) [23] and the Subjective Experience of Negative Symptoms (SENS) [24]. Items are scored on a 4-point scale. In the current analyses, CAPE dimensions of frequency of positive experiences (20 items) and negative experiences (14 items) were included (measured at baseline and 3-year follow-up), representing the person's perceived psychosis load over the lifetime (at baseline) or in the past three years (follow-up). A total score representing the mean of all items was calculated for each dimension (CAPE positive: range in siblings and comparison subjects: 0–2.5; CAPE negative: range 0–2.4).

### Other measures

At baseline and at follow-up, the short form of the Wechsler Adult Intelligence Scale (WAIS) – III was assessed for an indication of intellectual functioning, and included the following tests: ‘Block Design’, ‘Digit Symbol’, ‘Arithmetic’ and ‘Information’ [25], [26]. The WHOQOL-BREF [27] was used at baseline and at follow-up to assess four domains of quality of life (1) *physical health*, (2) *mental health*, (3) *social relationships* and (4) *environment*. At baseline, the Premorbid Adjustment Scale (PAS) [28] was administered. The PAS is a rating scale that includes measures of social isolation, peer relationships, functioning outside of the family, and school functioning at 3 age periods (up to age 12 [4 items], 12–15 [5 items], 16–18 years [10 items]). Validity, interrater reliability and internal consistency have been found to be high [29], [30]. An overall score based on the three age periods was created with a sample range from 0 (healthiest adjustment) to 5 (lowest adjustment).

### Follow-up

Healthy comparison subjects and siblings were eligible for follow-up. Of these, 78% (n = 1272) were assessed at 3-year follow-up (healthy comparison subjects: 78%, n = 462; siblings: 77%, n = 810). Measures of cannabis use at follow-up reflected use over the interval between baseline and follow-up. Ratings of CASH, SCAN, SIS-R and CAPE at follow-up reflected the period between baseline and follow-up. Mean follow-up was 3.3 years (SD  = 0.5).

### Transition

Transition from health to psychotic disorder was defined as (i) onset of non-affective psychotic disorder in individuals without psychotic disorder (DSMIV 295, 297, 298) and without psychotic affective disorder at baseline [n = 11; 7 with 295, 3 with 298, 1 with documented psychotic illness who refused follow-up], (ii) onset of affective disorder or other non-psychotic disorder with evidence of psychotic symptoms rated of at least “considerable” or “severe” quality (or equivalent) on the CASH, PANSS or SCAN in individuals without baseline affective disorder or other non-psychotic disorder and without evidence of psychotic symptoms rated of at least “considerable” or “severe” quality at baseline (n = 0). Individuals who refused to be seen at follow-up were queried about mental health and contacts with mental health services. Participating relatives of refusing participants also provided information.

### Analysis

Analyses were conducted using Stata, version 12 [31]. Analyses focused on the siblings (n = 1057 at baseline and n = 810 at follow-up) and healthy comparison subjects (n = 589 at baseline, n = 462 at follow-up). The dependent variable in the analyses was transition to psychotic disorder. Standard errors were corrected for hierarchical clustering of the data at the level of the family (clustering of siblings in the same family) or, when applicable, for clustering at the two levels occasioned by clustering of individuals in the same family and of repeated measures within the same person, using the Stata routines of *cluster*, *xtreg* or *xtmixed*, as appropriate.

Associations were expressed as the odds ratio from the logistic regression model (dichotomous transition outcome) or the regression coefficient (B) from multilevel random regression models (continuous variables). All analyses were *a priori* adjusted for age and sex. Comparisons between transition and non-transition status were additionally adjusted for sibling high risk status, in order to verify whether transition and non-transition differed in key variables independent of sibling high risk status.

In order to validate transitions, a comparison was made between transition and non-transition status with respect to key baseline variables as well as with respect to changes from baseline to follow-up. We thus expected that those who would make a transition to psychotic disorder would display more developmental impairment and higher levels of psychometric risk indicators at baseline (as measured with the CAPE, WHOQOL, PAS and WAIS). Differences in change from baseline to follow-up were examined in an *xtmixed* model of a repeated measure, whilst fitting an interaction between measurement occasion and transition status. Stratified associations were derived by linear combination from the model containing the interaction using the Stata *margin* command. The population attributable fraction associated with proxy environmental and genetic exposures was calculated using the *cc* command in Stata, and defined as the reduction in incidence that would be observed if the population were entirely unexposed, compared with its current exposure pattern.

## Results

### Sample and attrition

At baseline, the risk set consisted of 589 healthy comparison subjects and 1057 siblings. Baseline demographic characteristics are shown in table 1. Of these, respectively 462 (men: 44%, mean age: 34.2 years, sd  = 10.6) and 810 (men: 44%, mean age: 30.5 years, sd  = 7.9) were seen at follow-up. Attrition was associated with male sex, urban environment and ethnic minority status, as well as with lower IQ and small differences in premorbid adjustment (Table 2). Attrition was not associated with age at baseline, CAPE positive or negative symptoms, cannabis use, childhood trauma and WHOQOL-BREF domains (small or non-significant differences; Table 2).

**Table 2:** Differences at baseline as a function of follow-up attrition.

		Mean or %	SD	n	F or χ2	p
Age at baseline	No follow-up	28.1	9.6	374	2.4	0.126
	Follow-up	28.9	9.2	1,272		
Male sex	No follow-up	51%		374	5.2	0.022
	Follow-up	44%		1,272		
Minority ethnic group	No follow-up	24%		374	37.5	<0.001
	Follow-up	12%		1,272		
Urban birth	No follow-up	41%		326	8.5	0.004
	Follow-up	32%		1,193		
Cannabis use	No follow-up	39%		369	0.4	0.547
	Follow-up	37%		1,272		
Early Trauma	No follow-up	50%		137	0.2	0.654
	Follow-up	52%		1,177		
CAPE positive	No follow-up	0.21	0.21	325	1.6	0.21
	Follow-up	0.20	0.18	1,156		
CAPE negative	No follow-up	0.50	0.37	325	3.1	0.077
	Follow-up	0.54	0.36	1,156		
WHOQOL physical	No follow-up	4.05	0.57	310	8.0	0.005
	Follow-up	4.15	0.53	1,174		
WHOQOL mental	No follow-up	3.85	0.55	309	1.0	0.326
	Follow-up	3.88	0.52	1,174		
WHOQOL social	No follow-up	3.86	0.76	309	1.2	0.282
	Follow-up	3.91	0.65	1,174		
WHOQOL environmental	No follow-up	3.89	0.57	309	26.7	<0.001
	Follow-up	4.06	0.47	1,174		
IQ	No follow-up	101.0	14.1	342	37.6	<0.001
	Follow-up	106.7	15.7	1,236		
PAS premorbid adjustment	No follow-up	1.20	0.63	347	4.9	0.027
	Follow-up	1.11	0.63	1,187		

### Transition

Those who made a transition to psychotic disorder were younger at baseline (transition: 22.9 years, sd  = 4.8; non-transition: 29.0 years, sd  = 9.2 years; F = 4.82, p = 0.028). Transition was not associated with sex (OR = 1.4, 95% CI: 0.4–4.7). The 11 transitions were characterized by higher baseline psychopathology (CAPE positive and negative domains), poorer WHO-QOL scores, lower IQ and poorer premorbid adjustment (Table 3). In addition, transitions also displayed greater increases in psychopathology from baseline to follow-up (CAPE positive and CAPE negative) and greater decreases in quality of life, with the exception of the environmental domain. Transition was not associated with changes in IQ (Table 3). One of the 11 individuals had a non-psychotic DSM-IV diagnosis at baseline (300.3 obsessive-compulsive disorder), in the absence of significant psychotic symptoms at interview.

**Table 3:** Key variable baseline values and change over time (baseline to follow-up) as a function of transition status.

		BASELINE					FOLLOW-UP									
					Baseline transition vs. non-transition					Change transition vs. non-transition						
										time x transition interaction^#^			Stratified change values*			
		mean	SD	n	B	p	mean	SD	n		p		B		p	
CAPE positive^A^	Non-Transition	0.2	0.2	1,147	0.34	<0.001	0.1	0.1	1,213	0.44	<0.001		−0.09		<0.001	
	Transition	0.5	0.3	10			0.9	0.7	8				0.35		<0.001	
CAPE negative^B^	Non-Transition	0.5	0.4	1,147	0.31	0.005	0.4	0.4	1,213	0.75	<0.001		−0.10		<0.001	
	Transition	0.8	0.3	10			1.5	0.5	8				0.65		<0.001	
WHOQOL physical^C^	Non-Transition	4.2	0.5	1,165	−0.69	<0.001	4.2	0.5	1,217	−0.68	<0.001		0.08		<0.001	
	Transition	3.5	0.7	10			2.9	0.9	8				−0.60		<0.001	
WHOQOL mental^D^	Non-Transition	3.9	0.5	1,165	−0.48	0.003	3.9	0.5	1,217	−0.70	<0.001		0.06		<0.001	
	Transition	3.4	0.6	10			2.7	0.9	8				−0.65		<0.001	
WHOQOL social^E^	Non-Transition	3.9	0.6	1,165	−0.37	0.076	3.9	0.7	1,216	−1.10	<0.001		0.04		0.062	
	Transition	3.6	0.8	10			2.4	1.2	8				−1.06		<0.001	
WHOQOL environmental^F^	Non-Transition	4.1	0.5	1,165	−0.75	<0.001	4.2	0.5	1,217	−0.23	0.159		0.12		<0.001	
	Transition	3.3	0.4	10			3.2	0.6	8				−0.10		0.527	
IQ	Non-Transition	106.8	15.6	1,227	−11.70	0.015	110.0	16.7	1,211	0.59	0.839		3.20		<0.001	
	Transition	93.8	16.6	10			101.1	19.4	8				3.80		0.198	
PAS premorbid	Non-Transition	1.1	0.6	1,177	0.62	0.001	−	−								
adjustment^G^	Transition	1.7	0.9	11			−	−								

All associations adjusted for age, sex and high-risk sibling status.

# Tests whether change over time is significantly different for transition versus non-transition participants.

* Change over time calculated separately for transition and non-transition participant.

A CAPE positive symptoms, higher scores indicating increased frequency of symptoms.

B CAPE negative symptoms, higher scores indicating increased frequency of symptoms.

C Quality of life − physical health, higher scores indicating better quality.

D Quality of life − mental health, higher scores indicating better quality.

E Quality of life − social relationships, higher scores indicating better quality.

F Quality of life − environment, higher scores indicating better quality.

G Premorbid adjustment, higher scores indicating more social isolation, fewer peer relationships, worse functioning outside family and worse school functioning.

### Environmental and genetic prediction of transition

The majority of high-risk siblings (68%) and healthy comparison subjects (60%) had been exposed to one or more environmental risks. The risk of transition in siblings (n = 9 out of 810, 1.1%) was higher than the risk in healthy comparison subjects (n = 2 out of 462, 0.4%; OR adjusted for age and sex  = 2.2, 95% CI: 0.5−10.3; Table 4). All transitions were associated with environmental exposure, compared to 65% of non-transitions (p = 0.014), with the greatest effects for childhood trauma (OR adjusted for age, sex and sibling status = 34.4, 95% CI: 4.4−267.4), cannabis use (OR = 4.1, 95% CI: 1.1, 15.4), minority ethnic group (OR = 3.8, 95% CI: 1.2, 12.8) and urban birth (OR = 3.7, 95% CI: 0.9, 15.4) (Table 3). The proportion of transitions in the population attributable to environmental risk (PAF), assuming causality, ranged from 28% for minority ethnic group, 45% for urban birth, 57% for cannabis use, 86% for childhood trauma, and 50% for high-risk sibling status (Table 4). Nine out of 11 transitions (82%) were exposed to both proxy genetic and environmental risk, compared to only 43% of non-transitions (p = 0.03; Table 5).

**Table 4:** Transition as a function of proxy environmental and genetic exposures.

		Non-transition		Transition		Odds ratio_adj_ *	95% CI	PAF ^#^
		n	%	n	%			
Minority position	Majority	1,117	88.5	7	63.6	3.8	1.2−12.8	28%
	Minority	145	11.5	4	36.4			
Urban birth	Non-urban	807	68.0	3	32.0	3.7	0.9−15.4	45%
	Urban	379	37.5	5	62.5			
Cannabis use	No use	798	63.2	3	27.3	4.1	1.1−15.4	57%
	Use	464	36.8	8	72.7			
Early trauma	No	921	78.9	1	11.1	34.4	4.4−267.4	86%
	Yes	247	21.2	8	88.9			
Any exposure	No	447	35.4	0	0.0	∞		
	Yes	815	64.6	11	100.0			
High risk group	Comparison subject	460	99.6	2	0.4	2.2	0.5−10.3	50%
	Sibling	802	98.9	9	1.1			

* Odd ratio's adjusted for age sex and high-risk sibling status.

# PAF  =  population attributable fraction, or the reduction in incidence that would be observed if the population were entirely.

unexposed, compared with its current exposure pattern.

∞ = OR is infinity due to zero denominator.

**Table 5:** Transition status as a function of exposure to proxy environmental (E) and/or genetic (G) exposures.

		Neither G nor E	G or E	G and E
Non-transition	n	184	539	539
	%	14.6	42.7	42.7
Transition	n	0	2	9
	%	0.0	18.2	81.8

Pearson chi2 (2)  = 7.0 Pr  = 0.030.

## Discussion

In order to assess the importance of environmental and genetic risk on transition from health to psychotic disorder, a prospective study of a cohort of individuals with average and high genetic risk was carried out. The findings suggest that the rate of exposure to any environmental risk in the population is very high, or semi-ubiquitous, and that transition from health to psychotic disorder is strongly dependent on such exposure. Thus, all environmental risk factors were associated with transition to psychotic disorder, with the greatest effect, in terms of both relative and attributable risk, for childhood trauma. Exposure to environmental risk did not vary as a function of genetic high risk status, suggesting absence of genetic control of environmental exposure, or gene-environment correlation. In those who made the transition to psychotic disorder, 82% were exposed to both proxy genetic and environmental risk, compared to only 43% of those who did not transition. This finding suggests that exposure to both genetic and environmental risk factors is necessary for transition, which is compatible with underlying gene-environment interaction. Careful prospective documentation therefore suggests most transitions can be attributed to powerful environmental effects operating against elevated background genetic risk.

### Incidence of transition

Johnstone and colleagues followed a cohort of 163 young adults at average and high genetic risk, of which about 12% made a transition to psychotic disorder within 2.5 years [7], representing a yearly transition rate of 4.9% [7]. The *yearly* transition rate in the current study was 0.34% for siblings (1.1%/3.3 years of follow-up), and 0.13% for healthy comparison subjects (0.4%/3.3 years of follow-up). Differences with the Edinburgh high risk study of Johnstone and colleagues [32] may be related to the fact that their “high-risk” denoted more familial loading (2 affected relatives), and that the mean age was younger (21 years). The incidence in the healthy comparison subjects of the current study (0.13%) appears high compared to the classic incidence estimate of schizophrenia (0.02%). However, a direct comparison is not valid as our outcome included all affective and non-affective psychosis, was restricted to a young age group and case identification did not depend on use of health care. Previous work has shown that the rate of psychosis, thus defined, is up to six times higher than typical estimates [33].

The binary concept of transition may be difficult to define [9]. However, in the context of the current study, transition was from health to psychotic disorder, a clear and valid qualitative contrast that can be assessed reliably in the context of a clinical follow-up. The comparisons between transition and non-transition add to the notion of a valid contrast, given pre-transition differences in premorbid adjustment and cognition, that did not further decline after onset, conform expectation [34], [35]. However, the finding that cognition does not decline after onset has not been undisputed [36]. In addition, individuals developing psychotic disorder over the follow-up period displayed higher non-specific indices of psychometric risk and maladjustment as measured with the CAPE and WHOQOL at baseline, conform the model of clinical staging [37]. Furthermore, transition resulted in substantial increases for these variables, indicating true clinical change.

### Comparison with previous work

[LOOSSER]The results are in line with previous research showing associations between several environmental risk factors and development of psychotic symptoms or psychotic disorder [1]–[4], particularly in those at high risk [5]. As “high-risk” in the current study was defined on the basis of higher than average genetic risk, rather that psychometric risk as observed in UHR samples [38] or samples with attenuated psychotic symptoms in the general population [39], comparison with previous work is limited. Both Habets and colleagues [40], as well as Welch and colleagues [41] showed that cannabis use was associated with differential impact on brain structures in individuals at familial high risk for schizophrenia, which Habets and colleagues furthermore did not observe in controls. Similarly, epidemiological studies have demonstrated that the impact of urbanicity on schizophrenia risk is greater in those with additional evidence of elevated genetic risk [21], [42], [43].

### Genetic risk and environment risk: ubiquitous?

Studies focussing on the nature and extent of molecular genetic risk for schizophrenia have provided “molecular genetic evidence for a substantial polygenic component to the risk of schizophrenia involving thousands of common alleles of very small effect” [44]. In other words, molecular genetic variation contributing to risk for schizophrenia can be considered ubiquitous and distributed. Interestingly, the current study, being one of the first to examine multiple environmental risks together, suggests that conceptually the situation with regard to environmental risks may be similar. Thus, most individuals in the population were exposed to one or more of the environmental risks included in this study, and most of the transitions were attributable to environmental risk factors, against a background of genetic risk (most of the transitions being siblings of higher than average genetic risk). Methodologically this is an important issue, as the impact of a risk factor on a disease outcome cannot be detected if the entire population is exposed, unless the population can be separated into those who are differentially susceptible. Given the very high rate of exposure to environmental risks, the results suggest that careful follow-up of samples of differential genetic risk for psychotic disorder may be necessary to examine the true impact of environmental risk factors.

The nature of the impact of the environmental risks examined in the current study requires further clarification. First, the sample was too small to examine to what degree the environmental risks acted additively or more-than-additively. Previous work in general populations samples suggest that relationships may be both additive [45] and more-than-additive [46], [47]. In addition, the focus was on postnatal risk factors, although pre-natal risks may also play an important role [48]–[51].

Second, the data are not informative as to when and how the environmental factors examined impact on development to increase risk, and whether environmental risks gave rise to enduring liability early in life, or acted as precipitants in individuals at higher than average genetic risk. The temporal focus of the current investigation was on transition from health to illness, and retrospective examination of environmental impact is methodologically challenging.

A remarkable finding was the very high relative and attributable risk associated with childhood trauma. Given the prospective nature of the investigation, bias associated with a “search for meaning” cannot explain the results, in agreement with a growing number of prospective analyses testing the relationship between childhood adversity on the one hand, and psychosis on the other [52]–[56]. The results confirm the need to urgently identify the nature and the mechanism of risk associated with early adversity, as well the clinical implications thereof [57].

### Methodological issues

Strengths of the study include careful prospective assessment and confirmation of control status by excluding those with a positive family history. Because of the relatively short follow-up period, the number of individuals in this study who transitioned to psychotic disorder was relatively small. Although some results were statistically conclusive, other analyses, for example risk associated with sibling status, were underpowered. As the sample will be seen again at six-year follow-up, amplification of the sample and more robust results will be possible, as well as more fine-grained testing of relationships between genetic and non-genetic risks. Another issue is selection, as the focus was on transition in siblings and healthy comparison subjects who had lived through a substantial period of risk. It cannot be excluded that the mix of risk factors impacting on transition varies as a function of age-at-onset, thus the results cannot necessarily be generalised to transitions from health to illness at all ages.
